# Influence of Polishing Treatment on the Topography of 3D‐Printed Provisional Resins: An In Vitro Study

**DOI:** 10.1155/ijod/6315172

**Published:** 2026-10-18

**Authors:** Yousif A. Al-Dulaijan, Abdulrahman Mohammad Aldossari, Abdulrahman Salem Bin-jabair, Hamed S. Alghamdi, Hanan Al-Johani, Abdulrahman A. Balhaddad

**Affiliations:** ^1^ Department of Substitutive Dental Sciences College of Dentistry Imam Abdulrahman Bin Faisal University Dammam Saudi Arabia iau.edu.sa; ^2^ College of Dentistry Imam Abdulrahman Bin Faisal University Dammam Saudi Arabia iau.edu.sa; ^3^ Oral and Maxillofacial Surgery Department College of Dental Medicine Umm Al-Qura University Makkah Saudi Arabia uqu.edu.sa; ^4^ Department of Restorative Dentistry Faculty of Dentistry King Abdulaziz University Jeddah Saudi Arabia kau.edu.sa; ^5^ Department of Restorative Dental Sciences College of Dentistry Imam Abdulrahman Bin Faisal University Dammam Saudi Arabia iau.edu.sa

**Keywords:** 3D printing, polishing, resin, roughness

## Abstract

Although 3D‐printing has been increasingly integrated in the fabrication of provisional restorations, the evidence regarding the impact of different polishing regimens on their topographical features is unclear. This in vitro study assessed the outcomes of different polishing treatments on roughness measurements of 3D‐printed provisional resin materials compared to that of a conventional polymethyl methacrylate (PMMA). Disc‐shaped specimens were obtained from a conventional PMMA (GC Unifast III) and two commercially available 3D‐printed provisional resin materials (Asiga DentaTOOTH, NextDent C&B MFH). Specimens were allocated into four polishing treatments: unpolished (control), conventional pumice, Optiglaze, or chairside polishing. Surface roughness parameters (Ra, Rz) were quantified with a noncontact optical profilometer after polishing treatment. Qualitative surface analysis was obtained with representative 3D topographical color maps. Ra and Rz data were analyzed with a generalized linear model and Bonferroni‐corrected post hoc tests (α = 0.05). Lowest roughness was identified in Asiga DentaTOOTH and highest in conventional PMMA (*p* < 0.001). Optiglaze and conventional pumice yielded lower roughness measurements than chairside polishing (*p* < 0.001), and unpolished resins showed the most unfavorable roughness outcomes (*p* < 0.001). In conclusion, Optiglaze demonstrated promising potential in the improvement of 3D‐printed provisional resin restorations’ surface integrity, thus enhancing prostheses biocompatibility and long‐term survival.

## 1. Introduction

Provisional dental restorations are indispensable in fixed and implant prosthodontic treatment procedures, serving as an intermediate resolution to maintain pulpal health, periodontal tissue recovery, positional stability, and adequate esthetics, thereby improving patient satisfaction and overall quality of life [1, 2]. Provisional restorations can be fabricated from a broad range of resin materials using either direct chairside or indirect laboratory fabrication approaches [3]. Conventionally, materials such as polymethyl methacrylate (PMMA) and bis‐acrylic resins have been employed for the fabrication of provisional crowns and fixed partial dentures by virtue of their ease of handling and cost‐efficacy [4]. Nonetheless, such materials present limitations, including stain susceptibility, polymerization shrinkage, poor surface texture, and reduced marginal fit [5, 6]. Based on the interim treatment length, these material‐related drawbacks may foster secondary caries and gingival inflammation, which hinder the success of the final restoration [5].

With the ongoing advancements of computer‐aided design and computer‐aided manufacturing (CAD/CAM) in dentistry, subtractive manufacturing milling‐based technologies have become gradually replaced with 3D printing approaches (i.e., additive manufacturing) [7]. 3D printing entails a stepwise layering approach of material deposition, generating final structures with complex geometries in a time‐efficient and environmentally sustainable manner, thus overcoming milling‐related drawbacks, namely, material waste, milling bur damage, and machining‐induced surface flaws [7, 8]. Moreover, 3D printing has effectively enabled the fabrication of assorted prosthetic and restorative dental applications from a diverse range of printable materials. Within the dental field, the leading 3D printing technology is vat‐polymerization (VAT), wherein a printable resin liquid is contained within a vat (i.e., tank) and then is solidified through polymerization. Based on the equipped polymerization source, VAT printers can be further subclassified into stereolithography (UV laser beam), digital light processing (DLP; UV projector), or liquid crystal display (light emitting diodes) [9].

Finishing and polishing procedures are frictional processes that eliminate restorative surface irregularities by means of progressively abrasive tools ranging from tungsten carbide burs and diamond rotary instruments to impregnated rubber tips, pastes, and glazes, thus generating a surface luster parallel to that of the natural dentition [10]. While conventional pumice was traditionally used as a cost‐effective method for polishing dental resins, its disadvantages primarily stem from the increased abrasiveness and risks associated with manual application, such as elevated pressure, speed, and temperature [11]. Likewise, chairside polishing kits present limitations that render increased roughness, heat generation, and disruption of marginal integrity [12, 13]. To overcome these drawbacks, improved surface texture may be achieved through resin‐based surface coating treatments, such as Optiglaze (GC America Inc., Alsip, IL, USA), a photopolymerized nano‐filled coating intended to improve stain and wear resistance [14]. The existing literature has investigated the effects of finishing and polishing protocols on color stability, microbial adhesion, and topographical properties of 3D‐printed provisional restorations [14–18]. Nevertheless, an optimal polishing method for 3D‐printed provisional restorations has not been established to date.

The advancements in CAD/CAM technologies led to the development of improved provisional printable resin formulations that can deliver superior color stability, surface texture, and accuracy [18, 19]. Variations in the printing parameters (build orientation, layer thickness, and support structures) and post‐processing conditions (polymerization time, polymerization unit, and washing solution) greatly impact the surface topography and mechanical performance of 3D‐printed dental prosthesis. Likewise, the finishing and polishing regimens largely determine the surface integrity, biofilm adhesion, stain resistance, strength, antagonistic wear, and tactile perception [20–24]. While manufacturers instruct specific printing and post‐printing parameters, the finishing and polishing protocols of 3D‐printed resins are mainly nonspecific [21, 25].

The roughness of a surface is defined as profile height variation in relation to a parallel plane within a confined zone. It is portrayed by a series of alternating wavelength peaks and valleys that are quantified by two‐ or three‐dimensional roughness parameters obtained with diverse profilometry devices [26, 27]. Among the 2D parameters are the roughness average (Ra), which signifies the arithmetic mean of the absolute vertical deviations (peaks and valleys) from a mean reference line within a sampling length, and the mean roughness depth (Rz), which represents the average vertical distance between the highest peak and lowest valley over multiple sampling lengths. Ra is not sensitive to individual peaks or valleys, whereas Rz is more sensitive to severe profile deviations [28, 29]. Thereby, a distinct analysis of both roughness parameters is essential to provide an overall analysis of surface height deviations, while Ra conveys a broad understanding of textural changes, it can mask extreme defects, whereas Rz specifically focuses on extreme peaks and valleys, thus exposing outliers. Previous studies reported that polishing contributed to changes in the Ra of the 3D‐printed provisional restorative materials [17, 18]; however, to date, there are no studies that have investigated the effects of diverse polishing regimens on the Rz parameter of 3D‐printed provisional restorative materials. Hence, the novelty of this research lies in the inclusion of recently introduced printable provisional resin materials, diverse polishing systems, and a dual roughness assessment approach.

Therefore, this study aimed to evaluate the changes in surface roughness parameters (Ra and Rz) of 3D‐printed provisional resin materials concurrent with different polishing treatments. The null hypothesis was that there would be no significant differences in surface roughness parameters (Ra and Rz) between the different resin materials or between different polishing protocols.

## 2. Materials and Methods

### 2.1. Study Design and Sample Size Calculation

Details on the experimental design and the tested materials are shown in Figure 1. One hundred forty‐four disc‐shaped specimens (*n* = 48 per resin material) with 10 × 2.5 mm dimensions were allocated to four subgroups according to the polishing protocol: unpolished, conventional pumice, Optiglaze, and chairside polishing. The sample size was determined as 12 specimens per group, similar to previous studies [30–32]. The power analysis was reassessed after the tests to verify the adequacy of the chosen sample size.

**Figure 1:**
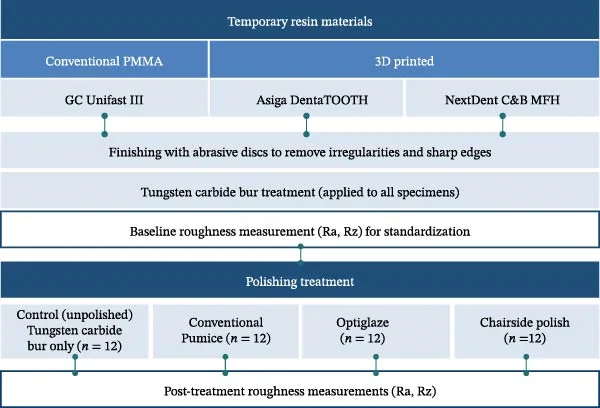
Experimental study design.

### 2.2. Sample Preparation

The conventional PMMA material (GC UNIFAST III, Tokyo, Japan) was prepared and mixed in accordance with the manufacturer’s recommendations. Two grams of powder was combined with 1 mL of liquid and manually mixed for 10–15 s using a plastic bowl and spatula. The resulting mixture was then packed into a circular mold measuring 10 × 2.5 mm, with a glass slide placed on each side to confine the material until complete setting was achieved.

For the 3D‐printed samples, STL file of a circular mold (10 × 2.5 mm) was used to print the sample. Utilizing an ASIGA MAX UV 385 printer (ASIGA, Erfurt, Germany) and a NextDent 5100 printer (NextDent B.V., Soesterberg, Netherlands), a total of 48 samples for ASIGA DentaTOOTH (ASIGA, Erfurt, Germany) and another 48 samples for NextDent C&B MFH (NextDent B.V., Soesterberg, Netherlands) were produced through 3D printing at a 45° printing orientation with a layer thickness of 50 µm [33, 34]. Both printers operate based on the DLP technology. After printing, all specimens were cleaned in 99.9% isopropyl alcohol for 10 min at a controlled temperature of 20°C and a relative humidity of 50%. Post‐curing was subsequently performed in accordance with each manufacturer’s instructions: ASIGA DentaTOOTH specimens were post‐cured in an Asiga Flash unit for 20 min at 60°C, whereas NextDent C&B MFH specimens were post‐cured in an LC‐D Print Box for 30 min at 60°C. Both post‐curing units operate at a wavelength of 405 nm. A summary of the material compositions and their respective printing parameters is provided in Table 1.

**Table 1:** Materials used, specifications, and printing procedures.

Material (brand name)	Conventional PMMA (GC UNIFAST III)	3D‐printed PMMA (Asiga DentaTOOTH)	3D‐printed PMMA (NextDent C&B MFH)
Composition	Methyl methacrylate, p‐Tolyldiethanolamine, Ethylene glycol dimethacrylate, Ethyl‐methyl methacrylate polymer, Polymethylmethacrylate, Barbituric acid derivative, Organic copper compound, Pigment	Bismethacrylate, Tetrahydrofurfuryl methacrylate, Diphenyl phosphine oxide	Micro‐filled hybrid with >60% of methacrylic oligomer
Printer	N/A	ASIGA MAX UV 385	NextDent 5100
Printing technology	N/A	Digital light processing Print orientation: 45°	Digital light processing Print orientation: 45°
Printing layer thickness	N/A	50 µm	50 µm
Post‐printing cleaning	N/A	Isopropyl alcohol 99.9% for 10 min at 20°C and a relative humidity of 50%.	Isopropyl alcohol 99.9% for 10 min at 20°C and a relative humidity of 50%.
Post‐curing conditions	N/A	Device: Asiga‐Flash Time: 20 min Temperature: 60°C Wavelength: 405 nm	Device: LC‐D Print Box Time: 30 min Temperature: 60°C Wavelength: 405 nm

Upon completion of the curing stage, a slow‐speed rotary instrument (MetaServ 250 Grinder–Polisher paired with a Vector Power Head; Buehler, Lake Bluff, IL, USA) was used to disconnect the support structures attached to the denture bases. The specimens were then uniformly finished using silicon carbide abrasive papers (3M Hookit 260L, ranging from 600 to 1200 grit; 3M Company, St. Paul, MN, USA) prior to the polishing protocol, with the entire finishing procedure performed consistently by a single operator [35].

### 2.3. Polishing Treatment

After the sample preparation, all the specimens were subjected to tungsten carbide finishing for 15 s at 10,000–15,000 rpm under water irrigation to mimic occlusal adjustments. Then, each group was subdivided into four subgroups according to the polishing protocol: unpolished, conventional pumice, Optiglaze, and chairside polishing. Details of the polishing protocol for each group are illustrated in Table 2. To ensure intra‐operator reliability and prevent inter‐operator variability, all polishing procedures were performed and calibrated by a single operator (Yousif A. Al‐Dulaijan), and all specimens were polished under water irrigation. For the unpolished group, specimens were subjected to a tungsten carbide cutter (No. 0620) with no further polishing protocol. For the conventional pumice group, the specimens were polished with the pumice slurry brush attached to a grading motors machine for 90 s, at 1500 rpm. While In Optiglaze group, Optiglaze was dispensed into a mixing dish, applied in one direction to the surface with a micro brush within 1 min of dispensing without air‐blowing, allowed to settle for 20 s, and light‐cured using light cure with wavelength 400–500 nm for 3 min. For all last group, a Shofu chairside polishing kit (AcryPoint, Shofu, Dental Corp, Menlo Park, CA, USA) was used, which contains three different grits (coarse, medium, and fine). From coarse to fine, the specimens were polished for 1 min at 10,000 rpm under water irrigation using a new polishing point per specimen.

**Table 2:** Polishing treatments details.

Group	Polishing system	Manufacturer	Composition	Protocol
Unpolished	Finishing only	Shofu Dental Corp, Menlo Park, CA	N/A	Tungsten carbide cutter (No. 0620) for 15 s at 10,000–15,000 rpm with no further polishing
Conventional pumice	Polishing slurry	Steribim Super, BEGO GmbH & Co KG; Bremen, Germany	N/A	• Tungsten carbide cutter (No. 0620) for 15 s at 10,000–15,000 rpm • Tungsten carbide cutter (No. 0620) for 15 s at 10,000–15,000 rpm
Optiglaze	OptiGlaze Clear HV (5 mL)	GC America Inc., Alsip, IL, USA	Methyl methacrylate (30%–60%), diphenyl(2,4,6‐trimethylbenzoyl) phosphine oxide (2.5%–5%), nanofillers and pigments (1%–5%)	• Tungsten carbide cutter (No. 0620) for 15 s at 10,000–15,000 rpm • OPTIGLAZE was applied with a micro brush and light cure with wavelength 400 to 500 nm for 3 min
Chairside polishing	AcryPoint chairside acrylic polishing kit	Shofu Dental Corp, San Marcos, CA	Bonded abrasives in silicone matrix	• Tungsten carbide cutter (No. 0620) for 15 s at 10,000–15,000 rpm • Trimmed with a dark gray coarse grit polisher (No. 0426) for 1 min, 10,000 rpm • Finished with a brown medium grit polisher (No. 0427) for 1 min, 10,000 rpm • Polished with a light gray fine grit polisher (No. 0428) for 1 min, 10,000 rpm

### 2.4. Roughness Measurement

The surface roughness (Ra, μm) and (Rz, μm) after each polishing protocol were recorded from the surface using a noncontact profilometer (Contour GT‐K1 optical profiler; Bruker Nano, Tucson, AZ, USA). Each specimen was scanned with a scanning area of 639 × 479 μm, lateral resolution of 0.33 μm, vertical resolution of <0.1 nm, and magnification of ×20 [30]. The average of three readings from each specimen was captured to record the final Ra and Rz values of each sample.

### 2.5. Statistical Analysis

Data normality was confirmed with the Shapiro–Wilk, kurtosis, and skewness tests. Considering that the Ra and Rz data did not violate the normality assumption, they were assessed using a generalized linear model with normal distribution and identity link function. Bonferroni‐corrected post hoc tests were used to analyze significant pairwise comparisons. All statistical analyses were conducted with a statistical software program (SPSS Statistics, v30.0, IBM Corp, Chicago, IL, USA) with a significance level set at α = 0.05.

## 3. Results

### 3.1. Roughness Measurements

Table 3 lists the results of generalized linear model; Ra was significantly affected by resin type (*p* < 0.001, η_p_^2^ = 0.845), polishing treatment (*p* < 0.001, *η*_p_^2^ = 0.949), and their interaction significant effects on Ra (*p* < 0.001, *η*_p_^2^ = 0.895). Descriptive statistics for Ra are depicted in Table 4 and illustrated in Figure 2. Irrespective of polishing treatment, significantly greater Ra values were identified among conventional PMMA, and lowest Ra were among the Asiga DentaTOOTH resins (*p* < 0.001). Regardless of resin type, Optiglaze and conventional pumice yielded lower Ra values (*p* < 0.001), whereas unpolished resins exhibited significantly increased Ra values, followed by chairside polishing (*p* < 0.001). No differences were observed between Optiglaze and conventional pumice, notwithstanding resin type (*p* = 0.807). Among unpolished specimens, conventional PMMA demonstrated considerably higher Ra followed by NextDent C&B MFH resins (*p* < 0.001). No differences were detected between Ra values of different resins polished with pumice (*p* ≥ 0.989). When chairside polished and Optiglaze were used, conventional PMMA showed higher Ra compared to Asiga DentaTOOTH (*p* < 0.001). Similar Ra values were detected across different polishing treatments among conventional PMMA (*p* ≥ 0.078) and NextDent C&B MFH resins (*p* ≥ 0.325). Conversely, Asiga DentaTOOTH resins polished with Optiglaze exhibited significantly lower Ra than that of chairside polished specimens (*p* = 0.018). Irrespective of the polishing treatment, greatest Ra values were found among the conventional PMMA, and lowest Ra were among the Asiga DentaTOOTH specimens (*p* < 0.001).

**Figure 2:**
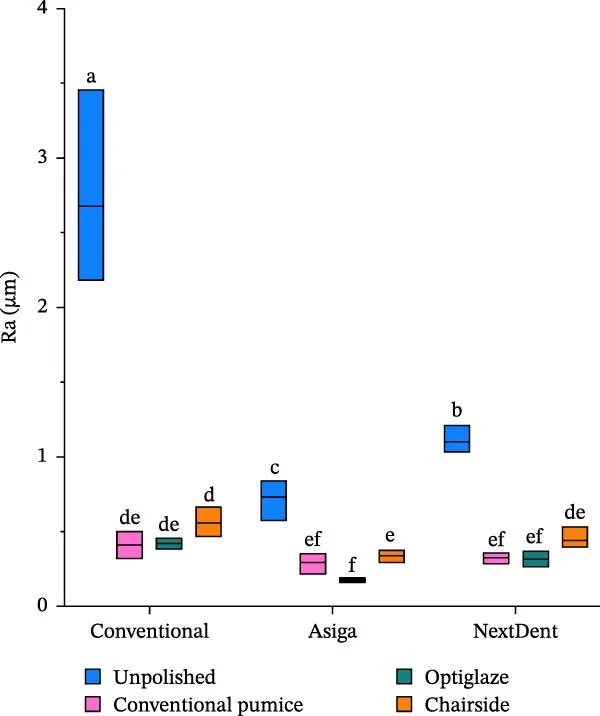
Ra roughness measurements of provisional resins subjected to different polishing regimens. Different letters denote differences between resin‐polishing treatment pairs (*p* < 0.05).

**Table 3:** Results of generalized linear model for surface roughness Ra parameter.

Outcome	Source of variation	Type III Wald Chi‐Square	df	*p*‐Value	*η*_p_^2^
Ra	Resin	785.396	2	<0.001	0.845
	Polishing treatment	2706.26	3	<0.001	0.949
	Resin × polishing treatment	4713.23	11	<0.001	0.895

**Table 4:** Descriptive statistics (mean ± standard deviation) of Ra values (µm) of provisional resins with different polishing methods.

Polishing treatment	Resin type			Pooled polishing treatment
	Conventional PMMA	Asiga DentaTOOTH	NextDent C&B MFH	
Unpolished	2.68 ± 0.38^A^	0.73 ± 0.08^C^	1.1 ± 0.06^B^	1.5 ± 0.88^a^
Conventional pumice	0.41 ± 0.06^DE^	0.29 ± 0.04^EF^	0.33 ± 0.02^EF^	0.34 ± 0.07^c^
Optiglaze	0.42 ± 0.03^DE^	0.17 ± 0.01^F^	0.31 ± 0.04^EF^	0.3 ± 0.11^c^
Chairside	0.56 ± 0.06^D^	0.34 ± 0.03^E^	0.44 ± 0.04^DE^	0.45 ± 0.1^b^
Pooled resins	1.02 ± 0.99^a^	0.38 ± 0.22^c^	0.55 ± 0.33^b^	—

*Note:* Different superscript uppercase letters represent differences between resin‐polishing treatment pairs, different superscript lowercase letters represent differences between pooled resin or pooled polishing treatments (*p* < 0.05).

Figure 3 demonstrates the 3D topographical color maps for all groups.

**Figure 3:**
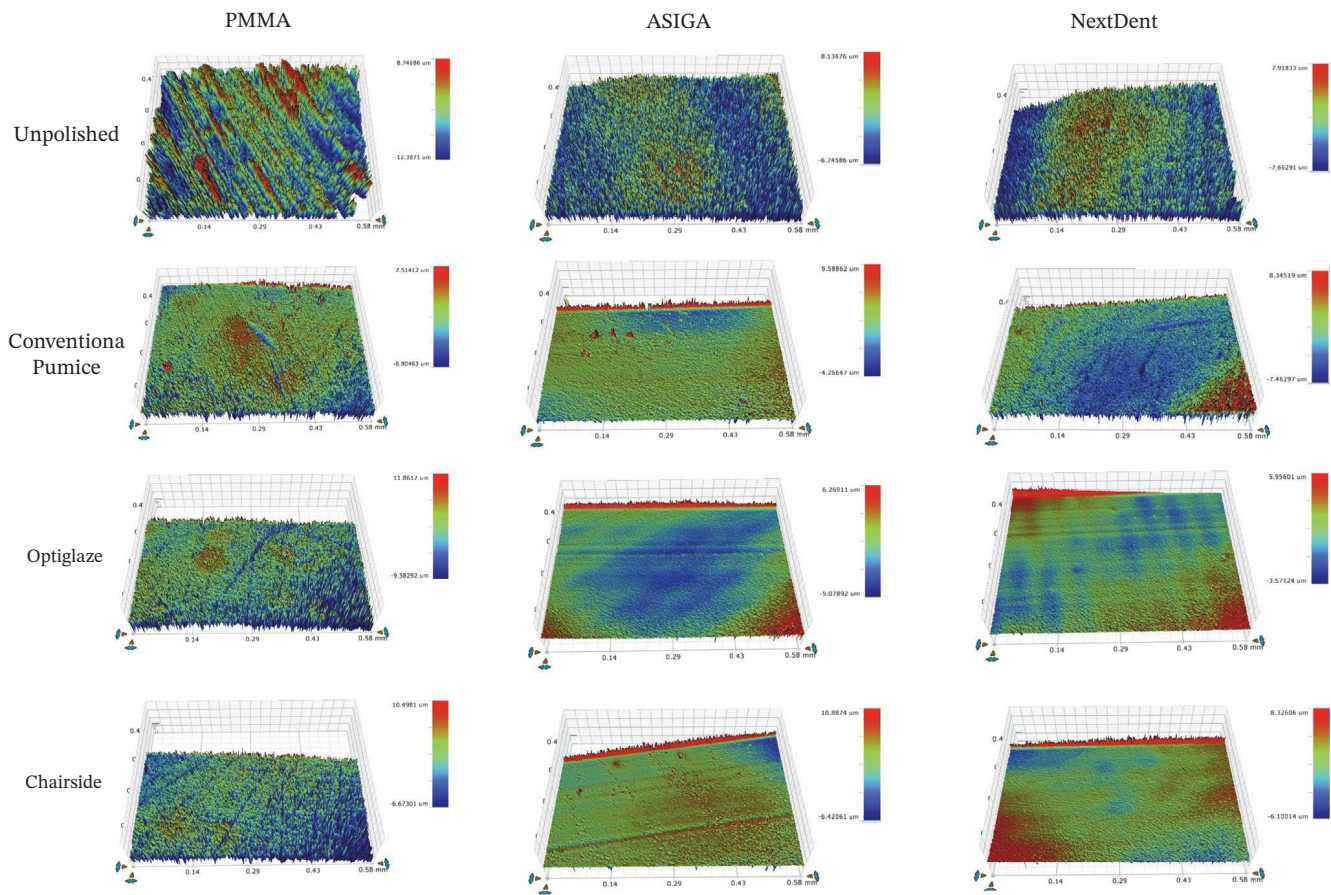
Representative 3D topographical color map for the investigated provisional resins subjected to different polishing treatment. Minor topographical deviations are represented by the blue color, moderate deviations by the green color, and severe deviations by the red color.

Table 5 presents the results of generalized linear model; Rz was significantly impacted by resin type (*p* < 0.001, *η*_p_^2^ = 0.445), polishing treatment (*p* < 0.001, *η*_p_^2^ = 0.667), and their interaction significant effects on Rz (*p* < 0.001, *η*_p_^2^ = 0.796). Descriptive statistics for Rz are listed in Table 6 and illustrated in Figure 4. Regardless of the polishing regimen employed, Asiga DentaTOOTH resins displayed significantly lower Rz values than that of conventional PMMA and NextDent C&B MFH resins (*p* < 0.001), while no differences were observed between conventional PMMA and NextDent C&B MFH resins (*p* = 0.978). Irrespective of resin type, considerably higher Rz were detected among unpolished specimens followed by chairside polished specimens (*p* ≤ 0.004), whereas no differences were identified between conventional pumice and Opitglaze polishing protocols (*p* = 0.331). Among unpolished specimens, conventional PMMA exhibited considerably higher Rz than both NextDent C&B MFH and Asiga DentaTOOTH (*p* < 0.001). Conversely, conventional pumice and Opitglaze treatments fostered similar Rz values across all resin types (*p* ≥ 0.999), and chairside polishing yielded considerably higher Rz in NextDent C&B MFH than other resin types (*p* < 0.001). Conventional PMMA and Asiga DentaTOOTH demonstrated similar Rz values throughout all polishing regimes (*p* ≥ 0.999), while NextDent C&B MFH showed higher Rz when chairside polished compared to other polishing methods (*p* < 0.001). Regardless of the polishing treatment, the lowest Rz values were detected in Asiga DentaTOOTH (*p* < 0.001).

**Figure 4:**
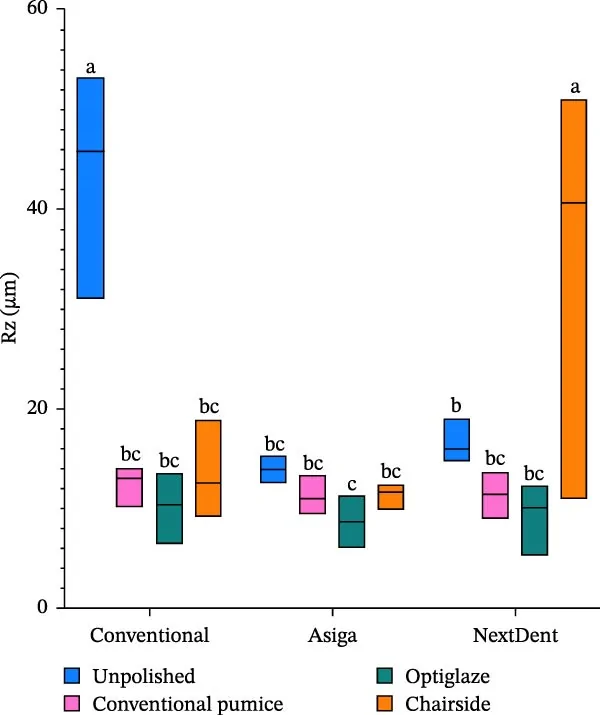
Rz roughness measurements of provisional resins subjected to different polishing regimens. Different letters represent differences between resin‐polishing treatment pairs (*p* < 0.05).

**Table 5:** Results of generalized linear model for surface roughness Rz parameter.

Outcome	Source of variation	Type III Wald Chi‐Square	df	*p*‐Value	*η*_p_^2^
Rz	Resin	115.383	2	<0.001	0.445
	Polishing treatment	288.834	3	<0.001	0.667
	Resin × polishing treatment	965.622	11	<0.001	0.796

**Table 6:** Descriptive statistics (mean ± standard deviation) of Rz values (µm) of provisional resins with different polishing methods.

Polishing treatment	Resin type			Pooled polishing treatment
	Conventional PMMA	Asiga DentaTOOTH	NextDent C&B MFH	
Unpolished	45.88 ± 8.88^A^	13.99 ± 0.7^BC^	16.03 ± 1.22^B^	25.3 ± 15.62^a^
Conventional pumice	13.08 ± 1.0^BC^	11.08 ± 1.16^BC^	11.45 ± 1.47^BC^	11.87 ± 1.48^c^
Optiglaze	10.48 ± 2.1^BC^	8.76 ± 1.75^C^	10.16 ± 1.88^BC^	9.8 ± 2.01^c^
Chairside	12.59 ± 2.7^BC^	11.67 ± 0.73^BC^	40.7 ± 13.07^A^	21.66 ± 15.58^b^
Pooled resin	20.51 ± 15.54^a^	11.38 ± 2.19^b^	19.59 ± 14.08^a^	—

*Note:* Different superscript uppercase letters represent differences between resin‐polishing treatment pairs, different superscript lowercase letters represent differences between pooled resin or pooled polishing treatments (*p* < 0.05).

## 4. Discussion

The present study findings revealed significant differences in the Ra and Rz roughness outcomes of 3D‐printed resins subjected to different polishing treatments. Moreover, dissimilarities were identified in the roughness values between dissimilar resin materials exposed to the same polishing regime. Therefore, both formulated null hypotheses are rejected herein.

Roughness is a clinically significant characteristic of restorative dental materials and is correlated with optical, biological, and mechanical considerations [23, 24, 28, 36]. It can be quantified by contact or noncontact profilometry, considering the former employs a stylus tip that potentially triggers surface damage interfering with roughness readings; thereby, noncontact optical profilometry was selected to obtain accurate roughness measurements herein [27, 29, 37]. Existing literature has verified the roughness values of sound enamel structure to range between 0.45 μm ≤ Ra ≤ 0.65 μm [38–40]; therefore, the current findings indicate that the 3D‐printed provisional resins tested in this study exhibited surface roughness parallel to that of natural enamel structures. Nevertheless, previous studies have established clinically relevant roughness thresholds, wherein Ra ≤ 0.2 μm is essential to prevent microbial adhesion [36], Ra ≤ 0.5 μm warrants reduced tactile perception [23], and decreased wear of antagonistic enamel is associated with Ra values less than 1.5 μm [24].

In the present investigation, all non‐polished materials surpassed clinically acceptable Ra roughness thresholds with values ranging between 0.73 and 2.68 μm. Additionally, among the treated groups, with the exception of Asiga DentaTOOTH coated with Optiglaze (Ra = 0.17 μm), all recorded Ra values exceeded the biofilm adhesion threshold above, ranging from 0.29 to 0.56 μm (Table 4). Nevertheless, a significant improvement in surface texture was detected after polishing treatments across all tested materials, irrespective of the implemented polishing protocol. Moreover, significant roughness differences were identified between the tested materials notwithstanding the polishing treatment, with substantially higher Ra found in conventional PMMA than that of 3D‐printed resins.

In the present study, the implemented printing parameters employed for the fabrication of the 3D‐printed specimens were standardized and were selected based on studies reporting improved marginal fit, precision, and fracture resistance among 3D‐printed provisional restorations with a 45° printing orientation and 50 µm layer thickness [33, 34]. Thereby, the observed disparities in Ra findings may be attributed to differences in the chemical composition, microstructure, or filler loading. The conventional PMMA (GC Unifast III) is a self‐cured acrylic resin without filler reinforcement, produced through a proprietary polymer processing method developed by GC Corp known as Surface Uniformity Revolutionary Fixation (SURF) in which the polymer particles are uniformly coated with a pigment to create homogeneous, bubble‐free mixtures. Asiga DentaTOOTH is comprised of a urethane dimethacrylate‐based formulation that does not include filler loading [41], whereas the composition of NextDent C&B MFH is fortified with methacrylic oligomers by >60% (Table 1). Furthermore, increased filler concentration, geometry, and size largely contribute to greater surface irregularities [42], which may justify the greater roughness findings of unpolished GC Unifast III and NextDent C&B MFH specimens. In terms of Ra changes, the influence of the investigated polishing treatments was relatively comparable among conventional PMMA and NextDent C&B MFH materials, which could bode promising potential for their clinical suitability. Nonetheless, Asiga DentaTOOTH specimens polished with Optiglaze demonstrated notably superior surface texture compared to specimens polished chairside; thus, it can be inferred that the implemented chairside polishing regime in this study is considered overly aggressive for 3D‐printed provisional restorations fabricated from Asiga DentaTOOTH materials. Although the observed Ra findings were in the range of those reported for similar materials [43, 44], they exceeded the roughness values reported in other studies [45], ostensibly due to dissimilar polishing regimens combined with the inclusion of a simulated aging process therein.

The depth of roughness variabilities can be determined through the measurement of the Rz parameter, which is considerably impacted by scratches, surface flaws, and contamination [29, 37]. Similar to the aforementioned Ra findings, significantly lower Rz was found in the Asiga DentaTOOTH resins, whereas no Rz differences were revealed between conventional PMMA and NextDent C&B MFH resins. Additionally, chairside polishing substantially increased the Rz values, regardless of the tested resin material, while no Rz differences were verified between conventional pumice and Opitglaze polishing treatments across all resin materials. Among the polished specimens, NextDent C&B MFH resins polished chairside displayed exceedingly elevated Rz values (40.7 μm). However, considering the inherent operator dependency of chairside polishing as well as the high standard deviation detected in the chairside polished NextDent C&B MFH group, this indicates notable intragroup variability therein which should be acknowledged. Based on this finding, it can be implied that dispersed deep scratches stemmed from the chairside polishing regimen of NextDent C&B MFH resins, plausibly by virtue of the inconsistent wear patterns between the methacrylic oligomers and the surrounding resin matrix. Therefore, 3D‐printed provisional restorations fabricated from resins with high filler loading may benefit from less invasive polishing treatments, such as glazing pastes or nano‐filled resin coating agents.

When comparing the three polishing systems utilized in this study, the Optiglaze treatment yielded the most favorable results, thus endorsing the manufacturing claims regarding its ability to achieve high‐gloss and wear‐resistant resin surfaces in a time‐efficient manner. This has been corroborated in the literature, where interim CAD/CAM milled restorations coated with Optiglaze exhibited smoother surfaces, higher gloss, and improved color stability [14, 46, 47] by virtue of its low viscosity that serves a sealant mechanism wherein the protective coating penetrates into the surface fissures and scratches developed during fabrication generating an even, homogenous superficial layer. In contrast, the higher surface roughness caused by conventional pumice and chairside polishing could be the result of the subjective pressure of manual application and the abrasiveness of the polishing kits [11–13]. Thereby, based on the preliminary findings of the present study, Optiglaze coating of 3D‐printed provisional resin restoration may provide favorable surface texture, potentially reducing the biofilm retention and enhancing prosthesis longevity.

The current study has several limitations, such as the lack of a subtractively manufactured provisional resin group and 3D‐printed definitive resin materials. Another limitation in this study is that the pressure applied during the polishing procedures was not standardized or quantified. Although all specimens were prepared by a single trained operator using a fixed sequence, speed, and duration for each protocol, no calibrated load‐application device was employed, and minor variations in applied pressure among specimens cannot be excluded. Moreover, this study was conducted under standardized laboratory conditions that did not involve any hydrothermal aging, dynamic chewing simulation, or erosive and abrasive challenges. Thus, investigating the influence of polishing regimens after artificial aging may offer greater insights regarding the long‐term effects on the roughness changes of 3D‐printed provisional resins in terms of water sorption, hydrolytic interfacial degradation, and antagonistic wear. Likewise, the absence of microscopic characterization is a limitation in the present study that could have provided further interpretation of the surface textural changes. Future experimental study models should include an assessment of supplementary surface properties such as color stability, gloss, hardness, and microbial adhesion, as well as mechanical characterization to improve the clinical relevance of this research and optimize the polishing protocols of 3D‐printed provisional resins in clinical practice. Likewise, further studies should explore the effects of extended toothbrushing and chewing simulation on the long‐term roughness stability of Optiglaze‐coated 3D‐printed provisional restorations under abrasive circumstances.

## 5. Conclusions

This study concluded that the application of a surface coating agent (Optiglaze) to 3D‐printed provisional resin materials notably enhanced the surface texture. This study also confirmed that chairside polishing rendered pronounced deep surface irregularities in resin formulations with increased filler loading that may negatively impact the esthetic outcomes, microbial adhesion, and mechanical durability of the 3D‐printed prosthesis due to its confounding operator‐dependent variables.

## Author Contributions

Conceptualization: Yousif A. Al‐Dulaijan, Abdulrahman Mohammad Aldossari, and Abdulrahman Salem Bin‐jabair. Methodology: Yousif A. Al‐Dulaijan, Abdulrahman Mohammad Aldossari, Abdulrahman Salem Bin‐jabair, and Abdulrahman A. Balhaddad. Formal analysis: Hanan Al‐Johani. Investigation: Yousif A. Al‐Dulaijan, Abdulrahman Mohammad Aldossari, Abdulrahman Salem Bin‐jabair, and Hanan Al‐Johani. Resources: Hamed S. Alghamdi and Abdulrahman A. Balhaddad. Data curation: Yousif A. Al‐Dulaijan, Abdulrahman Mohammad Aldossari, Abdulrahman Salem Bin‐jabair, and Abdulrahman A. Balhaddad. Writing – original draft preparation: Hamed S. Alghamdi and Hanan Al‐Johani. Writing – review and editing: Hamed S. Alghamdi, Hanan Al‐Johani, and Abdulrahman A. Balhaddad. Supervision: Yousif A. Al‐Dulaijan, Hanan Al‐Johani, and Abdulrahman A. Balhaddad.

## Funding

This research received no external funding.

## Disclosure

All authors have read and agreed to the published version of the manuscript.

## Conflicts of Interest

The authors declare no conflicts of interest.

## Data Availability

The data supporting this study’s findings are available from the corresponding author upon reasonable request.
